# Psychological Support for Health Professionals: An Interpretative Phenomenological Analysis

**DOI:** 10.3389/fpsyg.2018.01816

**Published:** 2018-09-27

**Authors:** Eleonora Volpato, Paolo Innocente Banfi, Chiara Valota, Francesco Pagnini

**Affiliations:** ^1^IRCCS Fondazione Don Carlo Gnocchi, Milan, Italy; ^2^Department of Psychology, Università Cattolica del Sacro Cuore, Milan, Italy; ^3^Department of Psychology, Harvard University, Cambridge, MA, United States

**Keywords:** burnout, health care professionals, neuromuscular disorders, chronic respiratory disorders, interpretative phenomenological analysis

## Abstract

**Background:** The work of health care professionals (HCPs) exposes them to emotionally difficult situations, anxiety, suffering and death, so they are at risk of burnout.

**Objectives:** To describe HCPs’ experiences of a psychological support intervention and its influence on the daily work of a sample caring for patients with neuromuscular and chronic respiratory illnesses.

**Methods:** This exploratory, descriptive, qualitative study was carried out at the Respiratory Rehabilitation Unit of IRCCS Santa Maria Nascente, in Milan, Italy. Semi-structured interviews with a purposive sample of 10 HCPs were subjected to interpretative phenomenological analysis (IPA).

**Results:** Five related themes emerged: psychological support; repeating the experience; relationships; the role of homework; competences. HCPs perceived that the intervention influenced their daily life, giving them a secure base and a new perspective on their professional role.

**Conclusion:** Psychological support interventions may not be appropriate for all HCPs, but they may help some HCPs to handle the demands of a stressful work life. Further studies are needed to determine its efficacy in reducing stress and prevent burnout.

## Introduction

Previous studies of practicing physicians and nurses and those in training (Rudman and Gustavsson, 2012; Shanafelt et al., 2015b) and health care professionals (HCPs) in general (Moss et al., 2016) report that the prevalence of burnout, a work-related syndrome, has reached 60% (Bodenheimer and Sinsky, 2014). Other studies have reported prevalence figures ranging between 25 to 75% amongst healthcare workers (Shanafelt et al., 2002; Fahrenkopf et al., 2008). Burnout affects workers’ ability to carry out their professional role (Dyrbye et al., 2010; Shanafelt et al., 2011) as well as the quality of the services, safety and HCPs wellbeing (West et al., 2012). Some studies have investigated the association between burnout and physical diseases in HCPs and found that exhaustion and depersonalization are strongly associated with cardiovascular and musculoskeletal symptoms (Honkonen et al., 2006; Seidler et al., 2014; Mikalauskas et al., 2018). It is also important to note that working is amongst the determinants of mental health outcomes such as anxiety and depression (Andrea et al., 2009; Pinto-Meza et al., 2013). Factors such as long working hours, perceived workload, concerns over complaints and poor communication with or lack of reciprocity between HCPs and patients, caregivers and colleagues reduce job satisfaction (Devalk and Oostrom, 2007), sometimes with indirect consequences for family life (Gingras et al., 2010). In addition, professional turnover due to work-related stress causes staff shortages and instability in healthcare teams, which may compromise the balance of healthcare teams, engagement, and patient care (O‘Brien-Pallas et al., 2006; Hayes et al., 2012). Furthermore, little is known about the impact of the National Health Care System (NHS) and organizational leadership on burnout and satisfaction of both team works and individual professionals. Few studies have documented the relationship between individual HCPs and division as a critical component in professional satisfaction (Demmy et al., 2002; Shanafelt et al., 2015a, April). Recent studies of the Italian NHS have emphasized that the recent financial crisis has limited the hiring of new workers over several years and noted that the Italian NHS has an aging workforce that is exposed to a high number of job stressors including lack of role clarity and resources, time pressure and a bureaucratic apparatus (Bressi et al., 2009; Carta et al., 2017). These considerations are even more important if related to the disease severity treated. In particular, caring for persons affected by neuromuscular disorders and chronic respiratory disorders could be considered especially challenging and becomes more challenging as the disease progresses (Bertram and Tanzi, 2005). Neuromuscular diseases are characterized by progressive degeneration of motor neurons in the cortex, brainstem and spinal cord, which hinders voluntary muscle movements and leads to weakness. The onset is mainly sporadic and the spread of symptoms through the limbs and the bulbar and thoracic regions leads to significant functional and physical impairments (Sivak, 2016). Chronic respiratory diseases of the airways and other structures of the lung (e.g., asthma, chronic obstructive pulmonary disease, occupational lung diseases, and pulmonary hypertension) limit airflow to and from the lungs and contribute to progressive functional impairment and a gradual decline in quality of life (QoL) (Dal Negro et al., 2015). Patients with neuromuscular and chronic respiratory diseases often have to get used to using devices for non-invasive ventilation (NIV), percutaneous endoscopic gastrostomy (PEG), or radiologically inserted gastrostomy (RIG), tracheotomy and communication and so both HCPs and caregivers need to be adequately trained in the use and maintenance of these devices to ensure that they follow recommended procedures meticulously (Sahni and Wolfe, 2018).

Despite their importance, few empirical studies have been carried out on interventions aimed at preventing or reducing burnout. The most effective person-directed programs are usually characterized by cognitive and behavioral interventions (Awa et al., 2010) such as stress inoculation training, rational emotive therapy, cognitive restructuring or behavioral rehash. Relaxation techniques, stress management and mindfulness have also been used frequently in recent decades (Marek et al., 2017). On the other hand, very little attention has been payed to situational, group and organizational strategies that aim to eliminate or modify communication and environmental characteristics (Le Blanc et al., 2007). In a recent meta-analysis, West and their colleagues noted that no specific burnout interventions have been shown to be better than others, underlying the importance of both individual and organizational strategies, even if their combination has not been studied yet (West et al., 2016).

To our knowledge there are no studies about the implementation of a psychological intervention in HCPs who work with patients affected by neuromuscular diseases and/or respiratory chronic conditions in a rehabilitation setting. Moreover, there is limited information about HCPs’ perspectives on this kind of intervention. The study aims to provide an in-depth HCPs perspective of issues related to burnout, psychological support and its impact on their work life with people affected by neuromuscular diseases and chronic respiratory illnesses in a Respiratory Rehabilitation Unit.

## Materials and Methods

### Study Design

We used a qualitative approach, interpretative phenomenological analysis (IPA). Because IPA prioritizes the life world, it was the ideal approach for exploring HCPs’ experiences of the psychological intervention and its impact on their work life. Moreover, we collected accounts from members of all the healthcare professions and thus we collected information and accounts from multiple perspectives and levels of experience. It was hoped that this information could be used to improve the design of the intervention.

### Study Setting

The study and the related interviews were conducted at the Respiratory Rehabilitation Unit of the IRCCS Santa Maria Nascente, Fondazione Don Carlo Gnocchi, in Milan (Italy).

This study was approved by the Ethics Committee of the IRCCS Fondazione Don Carlo Gnocchi on 14th June 2015. Written informed consent was collected from all participants.

### Participants

In July 2016 all nurses, healthcare and social assistants, case managers, psychologists, medical doctors and speech therapists at the hospital were given information about the study, including information about arrangements to protect the confidentiality of participants’ data, and invited to participate. They were given a form which they were asked to use to indicate whether or not they consented to take part in the study. Non-respondents received reminder emails or phone calls one and 2 months later. Ten HCPs decided to join the study.

### Eligibility Criteria

Purposive sampling was used to recruit participants. The inclusion criteria were as follows: -age > 18 years old; -employed as a physicians, physiotherapists, nurses or care workers; and -employed at the HD Respiratory Rehabilitation Unit of the IRCCS Santa Maria Nascente of Fondazione Don Carlo Gnocchi, in Milan (Italy).

The information collected included sociodemographic information such as place and date of birth, gender, level of education, profession, residential arrangement, and marital status.

### Instruments

During the first assessment, we collected *sociodemographic information* (place and date of birth, gender, level of education, profession, residential arrangement and marital status).

*Semi-structured interviews* were carried out individually, in a private room free from the risk of interruptions and were scheduled at a time compatible with the interviewees’ professional responsibilities. The interviewer was a psychologist (CV), with experience in burnout issues, and training in the conducting of individual, semi-structured interviews. The interviews were conducted by a different psychologist from the one who conducted the experimental intervention (EV). Participants were informed during the consent process about the aim of the study and the importance of the interviews as a method of gaining insight into their experiences.

Semi-structured interviews were conducted at the end of the intervention, to explore participants’ self-observation skills and perception of their own potential and resources in the management of critical issues. The main topics explored were motivation for joining the study, whether the psychological support group had influenced their work life and personal relationships, their experiences in the support group and how they would describe these experiences to a colleague. Prompt questions were used to facilitate disclosure.

The interview protocol was evaluated beforehand by an expert panel to ensure face validity. IPA indicated that it was characterized by open and exploratory questions and focused on the interviewee’s lived experiences and understanding of his or her path in his or her context. The design of the interview protocol was informed by the literature dealing with difficulties in the health work environments and in patient-professional communication. The participants were asked to describe the parts of the intervention that had made an impact on them and those that had shown them something about their work’s experiences and emotions. Particular emphasis was placed on exploring the interviewee’s actions, thoughts and feelings regarding his or her job. A copy of the interview protocol is available from the corresponding author on request (cfr. **Box 1**). The study was carried out according to the principles of exploratory sequential design; quantitative and qualitative data were collected and analyzed sequentially, starting with a quantitative design and deepening the phenomenon with a qualitative one (Ivankova et al., 2006; Fetters et al., 2013).

**BOX 1** | The main interview topics.

***Questions***

1. What kind of meaning has taken the path for you?
2. What feelings and emotions did you experience during the sessions?
3. What were the strong points of this path in your opinion?
4. What were the weaknesses of this path?
5. What would you suggest to improve this path?
6. What do you think of the “homework” assigned at home?
7. What are the skills/competences you feel you have acquired?
8. What are the effects of this path on your working relationships?
9. What are the effects of this path on your personal/family relationships?
10. Why would you suggest to repeat the experience?

The interviews were audio-recorded, transcribed verbatim, and reviewed. In addition, field notes were made in order to capture non-verbal responses to questions.

### The Intervention

This was configured as a person-directed program (Awa et al., 2010) to improve HCPs’ skills in handling their work relationships, with a particular emphasis on managing relationships with patients and their families and on being aware of one’s own feelings and behaviors.

We were unable to find in the literature a structured protocol suitable for our purposes, so three psychologists held meetings with the management (e.g., head nurse, head physician, and coordinators) of the Unit at which the intervention was to be delivered in order to agree a protocol, objectives and success criteria for the intervention and discuss potential benefits and problems. Structured and policies information were gathered and the perceptions of the Unit’s managers were considered.

The psychological intervention, consisted of five sessions of 2–3 h each at approximately 15-day intervals. The content of sessions was as follows:

– First session: Education on burnout. The objective of the session was for participants to know and be able to recognize the characteristics of burnout.
– Second session: The objective was for participants to know and be able to recognize risk factors and the process of burnout in the healthcare context. The group worked to develop participants’ awareness of critical work situations and shared examples.
– Third session: The objective was for participants to learn coping techniques and strategies for managing and controlling specific components (physical, psychological, and relational) of burnout syndrome, with the wider aim of thus preventing burnout.
– Fourth session: The group worked on positive relational strategies aimed at improving the working atmosphere, increasing professional satisfaction and reorientation of future perspectives in a constructive dimension of sharing, improving everyone’s empowerment. The aim was to reduce the stress caused by dysfunctional managerial and relational models and to influence the perception of effectiveness of workers in contact with users.
– Fifth session: Group work on the importance of managing the consequences of burnout (physical, psychological, and relational).
– Individual support meetings with the psychologist were held at the request of participants or their category manager.

Throughout sessions the atmosphere was kept warm and friendly, realizing a space for participants’ experiences both in class and at their home practice. Rooms for the sessions were booked in advance, taking into account the respective wards. Particular attention was paid to improving participants’ tolerance for and ability to transform difficult emotions during work-related situations. We opted to use a group format rather than a one-to-one format because the group could be used as a forum for improving communication amongst HCPs and between them and their patients. The availability of social support at work is fundamental, especially in the adaptation and management of care (Le Blanc et al., 2007). Moreover, in work environments in which team work is very important, such as the context in which our participants worked, being part of a group gives one access to peers who can empathize with one over the stress one has to deal with; the group format can also lead to improvements in participants’ understanding of complex events and situations. The idea of group interventions is that participants are able to learn new skills in a safe environment, in the company of professional peers and will then be able to transfer these skills to their work context (Sandberg, 2001). Finally using a group format saves costs and time, which has a positive impact on patient satisfaction (Garman et al., 2002). In addition to attending scheduled session participants were required to do some homework (e.g., they were asked to read relevant literature or watch a video about the issues covered in the session; between two of the sessions they were asked to carry out brief guided relaxation on a daily basis).

### Analysis

Interpretative Phenomenological Analysis was used in order to obtain access to the HCPs’ daily work experiences and their perceptions of the psychological intervention in which they had participated. We also explored HCPs’ perceptions of their relationships with colleagues, patients and caregivers during daily work. IPA focuses on how people make sense of their own experiences and requires the researcher to collect detailed, idiographic, reflective accounts from participants (Smith and Osborn, 2004; Larkin et al., 2006). The researcher’s reflections are an essential part of the IPA process and the researcher should try to remain aware of his or her preconceptions, which are acknowledged as factors in the process (Finlay and Gough, 2008).

An ideographic approach was used. Initially, each transcript was read several times in succession to enable the researchers to familiarize themselves with the content and gain an overall impression of it. Interesting passages were highlighted and notes made in the margins. At first these notes reflected reactions to the text but they gradually became more specific; they could be descriptive, linguistic or conceptual. Therefore, the analysis began with a deepen investigation before the other cases were included and a general categorization emerged, paying attention to the richness of text passages and the expressed contents. Thus each interview was read several times and notes were made. On re-reading a transcript themes emerged and connections between them were identified. Finally, the themes were ordered coherently and a table created. This procedure was applied to all transcripts and final table was produced to aid interpretation and reinforce the links between the themes and the raw data. Conceptually, similar themes were place in close proximity and different or incongruent themes were proportionally posed at distance.

All analyses were performed twice, by two members of the research team working independently (EV; FP) and themes were identified using NVivo (Version 11, QRS International, Lancashire, England), a qualitative analysis software package (Morse and Field, 1995). To improve the reliability and validity of the data, discrepancies in theme categorization and coding were addressed by a third research team member (CV). The researchers discussed what the experiences the participants described really meant and tried to remain open to new ways of interpreting participants’ perceptions, their work life and the meaning they give to it. Finally, critical scrutiny of the analysis process, with the participants actively involved, was carried out in order to uncover the researchers’ preconceptions (Malterud, 2001).

## Results

### Sample Characteristics

Ten semi-structured interviews d with a mean duration of 20 min (*SD* = 3.51) were carried out between September and December 2017. Three participants dropped out, one due to time constraints and two because they moved work team.

The mean age of participants was 44 years (*SD* = 9.39 years); three were male and seven were female; six were married, three single and one was cohabiting. The professional role most heavily represented was physiotherapist (*n* = 4), the other roles represented were nurse (*n* = 2), healthcare assistant (*n* = 3), and physician (*n* = 1) (**Table 1**).

**Table 1:** Socio-demographic characteristics of the sample.

	Participants (n.10)
**Gender**	
Male	3/10
Female	7/10
**Age in years**	
M (SD)	44 (9.39)
**Education Level**	
Middle School	2/10
Upper Secondary School	2/10
Graduation	4/10
Specialization	1/10
Master/Ph.D.	1/10
**Marital Status**	
Unmarried/Maiden	3/10
Married	6/10
Cohabiting	1/10
**Profession**	
Careworker	2/10
Professional nurse	2/10
Physiotherapist	4/10
Doctor	1/10
Other	1/10

*M, mean; SD, standard deviation.*

### Qualitative Analysis

The results are organized in terms of the five themes that emerged: psychological support; repeating the experience; relationships; homework; improving and learning competences. These themes were closely related to each other. The main impact of the psychological intervention was on competences and relationships. It also enhanced participants’ access to both internal and external resources and improved their adaptation and relational skills.

**FIGURE 1:**
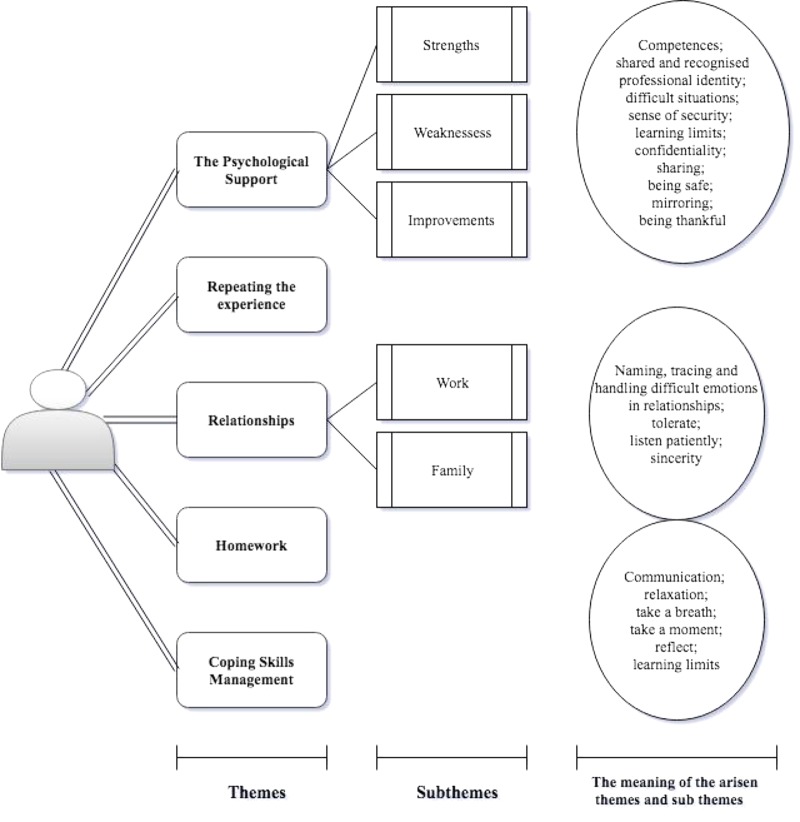
Diagram of themes and subthemes.

Subthemes were also identified, providing a more detailed picture of the themes (**Figure 1**). In particular, the first theme, the psychological support, allowed to identify strengths, limitation and possible areas of improvement of the intervention; whether the them about relationship favor the arisen of reflections related to the colleagues (work relationships) of spouses and children (family relationships). In **Figure 1**, moreover, are highlighted, enclosed in circles, the values, meanings and actions to which the participants have referred with respect to the respective themes and sub-themes.

#### Psychological Support

One finding that emerged was that participants considered the intervention group a source of psychological support for the multidisciplinary team (**Table 2**).

**Table 2:** Themes and subthemes arisen from the IPA analysis with illustrative quotations.

Themes	Subthemes	Illustrative quotations	Meanings
Psychological support	Strengths	*“I feel less lonely, both because there someone was interested in us, and because I realized that other workers and colleagues have stress problems similar to mine”* (ID14) *“... using techniques like the ones we were shown was new to me, but I realized that they can be useful in stressful situations...”* (ID2) *“The fact of being able to talk about the stress and ways of coping together with my colleagues… everybody contributed something to the discussion and then everything was needed to become aware of our work and difficulties. So being with my colleagues, sharing, I think that’s a strong point!”* (ID20) *“The strengths were the availability of the people who did the course, both at sessions and between sessions. They were available for individual sessions to go in to more detail on all the various topics”* (ID7)	Competences; shared professional identity; acknowledgment of fellow participants’ professional identities; difficult situations; sense of security; learning limits; confidentiality; sharing; being safe; mirroring; being thankful
	Weaknesses	*“It was too short...”* (ID15)	
	Improvements	*“Perhaps I would have more sessions, closer together”* (ID 14)	
Repeating the intervention		*“...if someone reports difficulties, like I did, why not offer it?”* (ID13)	
Relationships	Work	*“I have a better balance between work and home. I can also leave work at the right time”* (ID13)	Naming, tracing and handling difficult emotions in relationships; tolerate; listen patiently; sincerity
	Family		
Homework		*“I found it much more challenging (than the sessions) because it took longer and you really had to carve out space at home…”* (ID2) *“...useful but certainly difficult to do, but this is a subjective thing, because I find it difficult to let go of certain thoughts…”* (ID15)	Communication; relaxation; take a breath; take a moment; reflect; learning limits
Coping skills and stress management		*“When you are faced with a difficult situation, you have to find a place …you should turn off your phone and put yourself there, even for just a minute…”* (ID17) *“...developing more awareness…often when we’re stressed we just react to a situation and we don’t stop to think. Our judgment gets clouded.”* (ID14)	

Many participants felt that changing work unit and type of user was stressful. All of them described the demands place on them by patients as stressful, chaotic and arduous, due to their complexity. They also explained that the performance of tasks was made much more complicated by interruptions to deal with other priorities. They found the intervention useful, in particular the suggestions and guidance on using relaxation techniques, non-guided breath focus and awareness training, which helped them to take a different perspective and improved their problem-solving skills. They found the intervention to be effective for reducing burnout and, in particular, negative emotions and subjective distress as well as effects of the working environment. Nevertheless, they complained that the intervention was too short and suggested that there should be more meetings at shorter intervals to faster continuity and a greater sense of being supported.

Participants considered that the strengths of the intervention were the opportunity it gave them to share emotional experiences, the way in which it increased their attention to and awareness of the importance of their professional role, and the opportunity it gave them to reflect on relational issues with colleagues.

Other participants emphasized the importance of sharing a safe space at a particular time. All participants valued the opportunity to discuss difficult issues as a group and appreciated the individual meetings offered between sessions.

#### Repeating the Experience

Participants described the intervention as worthwhile and worth repeating. They said they would describe it to a colleague as a useful way of dealing with burnout and as an exciting experience. Participants said the intervention gave them increased awareness of their feelings, emotions and thoughts. They suggested that regular participation in a similar intervention could be a way of preventing or dealing with burnout, because of the attention paid in the intervention to improving problem-solving skills and skills for coping with stressful situations.

#### Relationships

Most participants said the intervention had a significant positive impact on their wellbeing and their relationships at work and with their family. Demands of adaptation, improved work-life balance and perseverance to handle different degrees of assignments were often mentioned by participants of all professional roles.

#### The Role of Homework

The homework assignments were valued as a continuation of the intervention and an opportunity to practice the strategies learned in sessions alone. However, some participants complained that it was difficult to find the time and space to do the homework.

#### Coping Skills Management

All the participants identified that they had learned new competences or improved existing competences thanks to the intervention. Three participants mentioned in particular that they had learned the importance of having a situation under control. They appreciated that the intervention had allowed them to reflect on the potentially different perspectives of other actors involved in a social situation as well as on actions and reactions, because they elicited their skill toward an empathic understanding. Moreover, they noted that the intervention had led to positive encounters with patients and their significant others, making them aware of their growing skills and contributing to their satisfaction. Negative encounters had prompted them to ask a colleague or the psychologist for help during the sessions. Another three participants stressed that the intervention had taught them the importance of being aware of the features of a situations and of reflecting before reacting. The intervention had helped them to realize that there are many different ways to categorize work experiences and that many of them are arbitrary.

All the participants stressed the importance of taking challenges as well as acute assignments as stimulating and analyzing the features of the situations, enlightening them as an opportunity to learn and talk together, in order to improve the quality of their life at work. Having a group containing people from different professions and with different identities emerged as important, because it was perceived to facilitate analysis of situations from different points of view and consideration of wider factors such as leadership, the health care system and bureaucracy.

## Discussion

The aim of this study was to create a rich and detailed account of HCPs’ experiences of a psychological intervention and its impact on their work life with people affected by neuromuscular diseases and chronic respiratory illnesses in a Respiratory Rehabilitation Unit. Analysis of interview data resulted in the emergence of five themes that are related to both the relevance and features of the intervention and the competences learned during it. In particular, we found that participants’ ability to balance demands and resources influenced their perception of their skills and the importance of their professional role. According to previous studies lead to different contexts, the findings of this study confirms the relevance of health based on the management of versatility and complexity, characterized by the compresence of practical, social, psychological and clinical factors and sensitive to a multidisciplinary integration (Iudici, 2015; Iudici et al., 2015). At the same time, it is important to note that the above-mentioned financial challenges have been worsened by demands for increases in productivity without a concomitant increase in time or resources. Efforts to improve service quality by introducing new metrics and requirements (e.g., electronic records) have often led healthcare executives to focus on external threats, which have led to mistrust and burnout amongst HCPs (Swensen et al., 2016). Further studies are needed to understand how these factors can be related to specific health care contexts such as that explored in this study.

Experiences of team work and self-efficacy as well as having the opportunity to share one’s emotions emerged as fundamental to people’s ability to take a different perspective on the relevance and quality of their work. Our results confirm the importance of providing support for staff involved in provided complex care and facing high demands from patients (Stacciarini and Troccoli, 2004). As mentioned in the Introduction, both neuromuscular and chronic respiratory diseases place a significant burden on HCPs as the physical and functional impairments imposed by the disease limit patients’ autonomy and make it more important that caregivers are adequately trained and reassured. Therefore, as emerged across the intervention, also the inter-dependence of these factors could impact on the relational and communication levels. Providing psychological support aimed at preventing burnout could be one way of helping HCPs to acquire strategies for handling difficult situations and could lead to an increase reflection in the professional setting. Furthermore, it could facilitate to access to peers who can empathize because they have experience of similar distressing situations (Salinsky and Sackin, 2000; Moss et al., 2016). Our results are consistent with previous research and confirm that listening to HCPs’ stories can provide a different perspective on their work and a deeper understanding of the daily demands placed on them, as well as highlighting that organizational changes and negative emotions such as anger or frustration can affect quality of care (Hallin and Danielson, 2007; Reader et al., 2008). In view of our findings, HCPs should be taught to recognize the risk factors for burnout syndrome and taught how to manage complex situations as well as the importance of seeking assistance when necessary (Mealer et al., 2009).

### Limitations and Strengths

Like most qualitative studies this exploratory study is limited by the small sample and the possibility that a self-selection bias reduced representation of subgroups with higher or lower levels of distress or different professional roles and identities, which might have influenced the themes that emerged (Hamberg et al., 1994). The findings may only transfer to the settings and professional roles we studied.

Despite these limitations the study has important implications for the training of health professionals who work with patients with neurodegenerative disorders. It indicates that it is important to give them the opportunity to share feelings of inferiority, mishaps and emotionally charged episodes which affect confidence with colleagues (Chew-Graham et al., 2002). Moreover, the limited resources needed to carry out the intervention means that it could be adapted for use in other healthcare settings.

It is also important to note that recognizing and managing symptoms of burnout could help to reduce absence from work due to mental and physical illness, which would have an indirect impact on healthcare costs.

## Conclusion

Detailed analysis of the outcomes of the psychological support intervention delivered to HCPs working in a Respiratory Rehabilitation Unit is beyond the scope of this research, but warrants investigation in future, in order to improve quality of care. Moreover, although there may be other ways to reduce stress amongst HCPs, our research suggests that one of the effects of improving their ability to handle patients with complex, neurodegenerative and chronic illnesses was a reduction in stress. Future studies may provide insight into the differences between professional backgrounds. Finally, it is important to note that ensuring that HCPs have the skills to remain and flourish in their jobs could be the starting point for larger scale improvements in the healthcare system.

## Author Contributions

EV and CV carried out the study. EV wrote the manuscript with support from CV. PB and FP supervised the project.

## Conflict of Interest Statement

The authors declare that the research was conducted in the absence of any commercial or financial relationships that could be construed as a potential conflict of interest.

## Abbreviations

HCPs: health care professionals
IPA: interpretative phenomenological analysis
NHS: national health care system
NIV: non-invasive ventilation
PEG: percutaneous endoscopic gastrostomy
QoL: quality of life
RIG: radiologically inserted gastrostomy.
